# Fallacious Theories

**Published:** 1883-11

**Authors:** 


### HOMŒOPATHIC PHYSICIAN,

A MONTHLY JOURNAL OF MEDICAL SCIENCE.

If our school ever gives up the strict inductive method of Hahnemann, we are lost, and deserve only to be mentioned as a caricature, in the history of medicine."—CONSTANTINE HERING.

Vol. III. NOVEMBER, 1883. No. 11.

### EDITORIAL.

FALLACIOUS THEORIES.—Ever since Hahnemann published the first edition of his *Organon*, there have been those of his followers who have sought to improve upon his teachings. And why? Were the practical results—the cures—made by following Hahnemann less numerous than was anticipated? We believe not; on the contrary, a goodly number of intelligent physicians have declared their cures increased in proportion as they became strict followers of Hahnemann. The closer and more precisely they observed his directions, as to "taking a case," as to dose, repetition, etc., the greater and grander were their cures. If this be so, why then this constant desire, ever present in the homœopathic school, to change, alter, or, as some would put it, *improve* Hahnemann's methods? The only reason we can find is that Hahnemann's method is an extremely laborious and difficult one; hence the desire is to render it easier. A most worthy object, if this rendering easy did not at the same time make it weak, faulty, and less able to do its work of curing. Unfortunately, so far, this has been the only result: less difficult, less worthy, quicker to apply, surer to fail.

The great and the distinctive feature of Hahnemann's method is that drugs are proved on the healthy individual *before* using them as curative agents. The symptoms thus produced are to be compared with those of the sick, and a similarity between the provings

(of the healthy) and the symptoms (of the sick) is the only reason for prescribing a drug. Not because Dr. A. recommends it, not because Dr. B. extols it as a specific, but only and solely because of this great similarity between the provings and the patients' symptoms, or, as we read in the *Organon:* * * "We ought to rely solely upon the morbid appearances, which medicines excite in healthy persons, [as] the only possible manifestation of the curative virtues which they possess." To find this curative agent is often a most arduous task, one that requires severe labor and intelligent judgment; chiefly so because of careless examining of patient; patient thoroughly examined, the task of prescribing becomes easy. To avoid this task new methods and fallacious theories have been advanced; at the present time three of these fallacies are conspicuously advocated:

1. The first we might mention as the pathological method, prescribing remedies for diseases. Such and such remedies are good for such and such diseases; all reference to symptoms is totally and loftily ignored. This method is very easy and very useless. Practitioners of this kind eke out a scant knowledge of Homœopathy by a liberal use of allopathic measures: the hypodermic syringe, the gonorrhœal injection, the pessary, and uterine washes; aloe and other purgative pills are kept on hand *to cure* constipation; and so on, through the list of allopathic folly. So much has been written on this subject of recent years that more is superfluous.

2. The second fallacy, now only too popular, is the using of unproven morbific products. An apostle of this departure wrote us some time ago: "*We get cures first and provings afterwards.* This gentleman doubtless considers himself a well-read homœopathist! Ignorance is, indeed, bliss sometimes. This method—the isopathic—does away with a grand feature of Hahnemann's method, drug proving and its corollary, the power of *prevision.* Hahnemann said, Prove a drug, and from its pathogenesis you will learn its curative powers. An isopathist says, Try a morbific product clinically and then you will know its value—an entire subversion and belittling of Homœopathy. In fact, it is not Homœopathy, it is something else, a new system with which Homœopathy has nothing in common. And, judging from the confession of one of its ablest champions, this method is unsuccessful and useless. Unscientific and useless, can stronger criticism be penned?

3. The third fallacy is a part of the one just noticed; it is this:

*potentiation makes a medicine homœopathic.* A drug that is not homœopathic, *i. e.*, is not the most like, the simillimum, to a set of symptoms in the third or thirtieth potency, becomes so if used in the cm or mm! Can greater folly be advocated? A drug is only homœopathic to a set of symptoms when its pathogenesis is similar to those symptoms. It is then homœopathic, no matter what potency it be used in. In which potency it will cure best is entirely another question. We believe the high will always, or *very nearly always*, cure best, but the lower potencies *do* cure. Some of our grandest cures have been made with them, and it is folly to assert that Homœopathy only begins with the higher and highest potencies. A wise homœopathist will not limit himself to any one potency, but will keep his mind unprejudiced to use whichever preparation seems to be best. If potentiation makes a medicine homœopathic, it must change it. Therefore, Aconite 3d, 30, and cm are three different drugs, useful for different cases, must have different pathogeneses, and should have different names. It is this fallacious teaching which bolsters up the twin heresy of isopathy. Syphilinum, it is said, is homœopathic, because it is potentized—very high.

All of these fallacious theories are grave departures from true homœopathic science. Let us remember old "Father Hering's last injunction, and take warning in time:

"If our school ever gives up the strict inductive method of Hahnemann, we are lost, and deserve only to be mentioned as a caricature in the history of medicine."

